# Determinants of response to inhaled extrafine triple therapy in asthma: analyses of TRIMARAN and TRIGGER

**DOI:** 10.1186/s12931-020-01558-y

**Published:** 2020-10-29

**Authors:** Dave Singh, Johann Christian Virchow, Giorgio Walter Canonica, Andrea Vele, Maxim Kots, George Georges, Alberto Papi

**Affiliations:** 1 Medicines Evaluation Unit, The University of Manchester, Manchester University NHS Foundation Trust, Manchester, UK; 2 Zentrum Für Innere Medizin, Abteilung Für Pneumologie, Universitätsmedizin Rostock, Rostock, Germany; 3 Center of Personalized Medicine: Asthma and Allergy, Humanitas University and Research Hospital IRCCS, Milan, Italy; 4 Global Clinical Development, Chiesi Farmaceutici SpA, Parma, Italy; 5 Respiratory Medicine Unit, University of Ferrara, University Hospital S.Anna, Ferrara, Italy

**Keywords:** Asthma, Pharmacotherapy, Long-acting β_2_-agonists, Long-acting muscarinic antagonists, Inhaled corticosteroids, Subgroup analyses, Eosinophils

## Abstract

**Background:**

A number of single-inhaler triple therapies are being developed for asthma, including the extrafine formulation of beclometasone dipropionate (BDP), formoterol fumarate (FF), and glycopyrronium (G). Given asthma is a heterogenous disease, we investigated whether the clinical response to the addition of the long-acting muscarinic antagonist component within inhaled triple therapy was impacted by a range of clinical characteristics.

**Methods:**

These were pre-specified and post-hoc sub-group analyses of TRIMARAN and TRIGGER, which were double-blind, 52-week studies comparing medium-strength (100/6/10 µg; TRIMARAN) and high-strength (200/6/10 µg; TRIGGER) BDP/FF/G with the respective BDP/FF strengths in adults with uncontrolled asthma and a history of ≥ 1 exacerbation. Co-primary endpoints were pre-dose forced expiratory volume in 1 s (FEV_1_) at Week 26 and the rate of moderate-to-severe exacerbations over 52 weeks. Key secondary endpoints: peak FEV_1_ at Week 26 and average morning peak expiratory flow over the first 26 weeks in each study, and severe exacerbation rate over 52 weeks (pooled data).

**Results:**

Baseline clinical characteristics (pre-specified analyses) had no consistent effect on the lung function improvements with BDP/FF/G. For the exacerbation endpoints, sub-groups with higher reversibility gained greatest relative benefit from BDP/FF/G versus BDP/FF. In post-hoc analyses with patients sub-grouped by screening blood eosinophil values, in TRIMARAN the greatest relative effect of BDP/FF/G versus BDP/FF on the lung function endpoints was in the ≤ 300 cells/µL group; in TRIGGER, eosinophil levels did not markedly influence the relative efficacy of BDP/FF/G versus BDP/FF. Eosinophil levels did not influence relative efficacy on moderate-to-severe or severe exacerbations.

**Conclusion:**

Overall, the relative efficacy of extrafine BDP/FF/G versus BDP/FF was not influenced by a range of clinical characteristics. However, some patient sub-groups gained additional benefit from BDP/FF/G for certain endpoints. In particular, for exacerbations the relative efficacy of BDP/FF/G was greater in more reversible patients.

*Trial registration *ClinicalTrials.gov: TRIMARAN, NCT02676076 (registered February 8, 2016, https://clinicaltrials.gov/ct2/show/NCT02676076?term=NCT02676076&draw=2&rank=1,); TRIGGER, NCT02676089 (registered February 8, 2016, https://clinicaltrials.gov/ct2/show/NCT02676089?term=NCT02676089&draw=2&rank=1)

## Introduction

Inhaled corticosteroids (ICSs) are the mainstay of therapy in asthma, either alone or in combination with a long-acting β_2_-agonist (LABA) [1]. However, many patients receiving an ICS/LABA combination have suboptimal control [2, 3]. In such patients, the addition of a long-acting muscarinic antagonist (LAMA) has been shown to improve lung function and reduce the risk of exacerbations [4], and is now a recommended treatment option (for example, Global Initiative for Asthma recommends tiotropium as an add-on treatment for patients at Step 4 or 5) [1]. However, this previously required the use of two different inhalers, of different design, requiring different inhalation technique, and often with different dosing regimens. This is not only inconvenient for patients and healthcare providers, but can lead to poor adherence and can increase the risk of suboptimal drug delivery due to incorrect device use, both of which can negatively impact outcomes [5–10]. In particular, in patients receiving ICS/LABA, initiating LAMA in a different inhaler is associated with high ICS discontinuation [11].

A number of single-inhaler triple therapies are therefore being developed for use in asthma. One of these contains an extrafine formulation (i.e., with mass median aerodynamic diameter < 2 µm) of the ICS beclometasone dipropionate (BDP), the LABA formoterol fumarate (FF), and the LAMA glycopyrronium (G) delivered via a pressurised metered-dose inhaler (pMDI). Such extrafine formulations result in improved deposition in the small airways [12], which is potentially important given that asthma patients with significant small airways dysfunction tend to have poorer asthma control and quality of life, and are at increased exacerbation risk [13]. TRIMARAN and TRIGGER were two double-blind, Phase III, 52-week studies comparing the efficacy and safety of medium-strength (100/6/10 µg; TRIMARAN) and high-strength (200/6/10 µg; TRIGGER) BDP/FF/G with that of the respective strengths of BDP/FF in patients with asthma that was poorly controlled on medium-dose (TRIMARAN) or high-dose (TRIGGER) ICS/LABA therapy, with TRIGGER including a third arm in which patients received open-label BDP/FF + tiotropium [14]. Overall, BDP/FF/G improved lung function [pre-dose and peak forced expiratory volume in 1 s (FEV_1_) and peak expiratory flow (PEF)] versus BDP/FF in both studies, with significant reductions in the rate of severe exacerbations in a pre-specified pooled analysis.

Asthma is a heterogeneous disease. Clinical phenotypes are defined as patients sub-grouped according to clinical characteristics that predict clinical outcomes, prognosis and/or response to treatment [15–17]. There is much interest in defining clinical characteristics in patients with asthma that influence the response to treatments, potentially facilitating personalised medicine [1, 15–17]. In the current manuscript, we therefore investigate whether the clinical response to the addition of the LAMA component within inhaled triple therapy in TRIMARAN and TRIGGER was impacted by a range of clinical characteristics. To do this, we used data from a series of pre-specified sub-group analyses of the co-primary and key secondary endpoints. Given eosinophil levels are being used to guide some therapy choices in asthma [1], we also report data from a series of post-hoc analyses by screening blood eosinophil counts.

## Methods

### Trial design and participants

The full design and inclusion/exclusion criteria of TRIMARAN and TRIGGER have been previously published [14]. Both studies recruited populations aged 18–75 years, inclusive, with a documented history of asthma for at least one year and diagnosed prior to the age of 40 years, pre-bronchodilator FEV_1_ < 80% of the predicted normal value, and a change in FEV_1_ of > 12% and > 200 mL 10–15 min after inhaling salbutamol 400 µg. Patients were to have uncontrolled asthma (Asthma Control Questionnaire-7 ≥ 1.5), a history of at least one exacerbation requiring treatment with systemic corticosteroids or an emergency department visit or in-patient hospitalization in the previous 12 months, and were receiving a stable dose of an ICS/LABA for at least four weeks prior to entry (TRIMARAN: medium ICS dose; TRIGGER high dose).

Patients who met the inclusion and exclusion criteria at screening had their asthma maintenance therapy switched to extrafine BDP/FF 100/6 µg in TRIMARAN and 200/6 µg in TRIGGER, two inhalations twice daily (BID) via pMDI for a 2-week open-label run-in period. At the end of the run-in period, patients were randomised to either continue BDP/FF (100/6 µg in TRIMARAN or 200/6 µg in TRIGGER) or to receive extrafine BDP/FF/G (100/6/10 µg in TRIMARAN or 200/6/10 µg in TRIGGER), all two inhalations BID via pMDI. A third treatment group was included in TRIGGER: open-label BDP/FF 200/6 µg two inhalations BID via pMDI plus tiotropium 2.5 µg two inhalations once daily via a soft mist inhaler; these patients are not included in the current analyses. Over the 52-week treatment period, patients attended visits at which data were collected from spirometry evaluations, with asthma exacerbations captured throughout the study. In addition, patients recorded their PEF pre-dose each morning and evening.

All patients provided written informed consent prior to any study-related procedure. The study was approved by the independent ethics committees at each institution, and was performed in accordance with the principles of the Declaration of Helsinki, and the International Conference on Harmonization notes for guidance on Good Clinical Practice (ICH/CPMP/135/95). The studies are registered with ClinicalTrials.gov: TRIMARAN, NCT02676076; TRIGGER, NCT02676089.

### Outcomes

The co-primary endpoints of TRIMARAN and TRIGGER were change from baseline in pre-dose FEV_1_ at Week 26, and the rate of moderate-to-severe exacerbations in each study. The key secondary endpoints were change from baseline in peak FEV_1_ at Week 26 and in average morning PEF over the first 26 weeks in each study, and the rate of severe exacerbations using data pooled from both studies. Severe exacerbations were defined as asthma worsening requiring treatment with systemic corticosteroids for at least three days, whereas moderate exacerbations were episodes of asthma worsening that were self-managed, defined in accordance with an American Thoracic Society/European Respiratory Society joint statement [18].

The current manuscript presents BDP/FF/G versus BDP/FF data for the co-primary and key secondary endpoints, to be able to evaluate the effect of the addition of an extrafine formulation LAMA in the same device (and so the BDP/FF plus tiotropium data from TRIGGER are not included). The two protocols pre-specified a series of analyses of these endpoints with patients sub-grouped according to age (< 65 versus ≥ 65 years), sex, body mass index (BMI; < 25, 25– < 30 and ≥ 30 kg/m^2^), smoking status (non- and ex-smokers; current smokers were excluded from the studies), asthma exacerbations in the year prior to entry (1 versus > 1), and reversibility (> 200–400 versus > 400 mL). In addition to these pre-specified analyses, we conducted a series of post-hoc analyses with patients sub-grouped by blood eosinophil levels (≤ 300 versus > 300 cells/µL) at screening, with severe exacerbations also analysed across the eosinophil continuum, both alone and in patients with a history of > 1 exacerbation in the previous year.

### Statistical methods

No adjustment for multiplicity was applied to the analyses in this manuscript. Pre-dose and peak FEV_1_ at Week 26 and average morning PEF over the first 26 weeks were analysed using a linear mixed model for repeated measures including treatment, visit, treatment by visit interaction and country as fixed effects, and baseline value and baseline by visit interaction as covariates (visit effect being replaced by inter-visit period effect for PEF), with data presented as adjusted (least squares) means, and adjusted mean differences between treatments with associated 95% confidence intervals (CIs) and p values. An unstructured covariance matrix was assumed. The number of asthma exacerbations over the 52-week treatment period was analysed using a negative binomial model including treatment, country and number of exacerbations in the previous year (1 or > 1) as fixed effects (the number of exacerbations was not included in the model for exacerbations sub-group analyses), and log-time on study as offset, and presented as adjusted asthma exacerbation rates, and adjusted rate ratios with 95% CIs and p values.

## Results

### Participants

Overall, 1149 patients were included in the intention-to-treat (ITT) population in TRIMARAN, with 1142 patients included in the TRIGGER ITT population receiving BDP/FF/G or BDP/FF. The mean ages of patients included in these analyses were 52.5 and 53.6 years in TRIMARAN and TRIGGER, respectively, with 38.4% and 39.9% male, mean BMI 27.9 and 28.5 kg/m^2^, and mean reversibility 502.8 and 490.0 mL. The mean number of exacerbations in the year prior to entry was 1.2 per patient in both studies, with 82.3% having a history of 1 exacerbation in TRIMARAN, and 17.7% a history of > 1; in TRIGGER these values were 77.9% and 22.1%. A total of 85.4% of patients in TRIMARAN were non-smokers, compared to 85.7% in TRIGGER. The analyses included all patients in the intention-to-treat populations in the BDP/FF/G and BDP/FF groups in the two studies (Table 1).

**Table 1:** Patient sub-groups in TRIMARAN and TRIGGER (intention-to-treat population)

	TRIMARAN		TRIGGER	
	BDP/FF/G 100/6/10 µg (N = 575)	BDP/FF 100/6 µg (N = 574)	BDP/FF/G 200/6/10 µg (N = 571)	BDP/FF 200/6 µg (N = 571)
Age, n (%)				
< 65 years	469 (81.6)	476 (82.9)	473 (82.8)	448 (78.5)
≥ 65 years	106 (18.4)	98 (17.1)	98 (17.2)	123 (21.5)
Sex, n (%)				
Male	221 (38.4)	221 (38.5)	212 (37.1)	244 (42.7)
Female	354 (61.6)	353 (61.5)	359 (62.9)	327 (57.3)
Body mass index, n (%)				
< 25 kg/m^2^	172 (29.9)	173 (30.1)	150 (26.3)	163 (28.5)
25–30 kg/m^2^	230 (40.0)	231 (40.2)	217 (38.0)	205 (35.9)
≥ 30 kg/m^2^	173 (30.1)	170 (29.6)	204 (35.7)	203 (35.6)
Smoking status				
Non-smoker	483 (84.0)	498 (86.8)	488 (85.5)	491 (86.0)
Ex-smoker	92 (16.0)	76 (13.2)	83 (14.5)	80 (14.0)
Asthma exacerbations in previous year				
1	473 (82.3)	473 (82.4)	439 (76.9)	451 (79.0)
> 1	102 (17.7)	101 (17.6)	132 (23.1)	120 (21.0)
Reversibility				
200–400 mL	259 (45.0)	279 (48.6)	299^a^ (52.4)	281 (49.2)
> 400 mL	316 (55.0)	295 (51.4)	271 (47.5)	290 (50.8)
Eosinophils				
≤ 300 cells/µL	345 (60.0)	351 (61.1)	334^a^ (58.5)	360^a^ (63.0)
> 300 cells/µL	230 (40.0)	223 (38.9)	236 (41.3)	210 (36.8)

*BDP* beclometasone dipropionate, *FF* formoterol fumarate, *G* glycopyrronium

^a^Data missing for one patient

### Pre-specified analyses by baseline characteristics

**Fig. 1:**
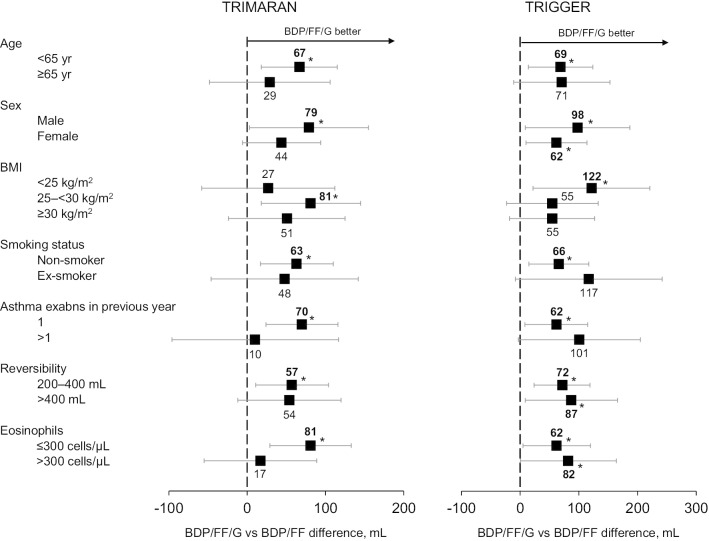
Change from baseline in pre-dose FEV_1_ at Week 26, adjusted least squares mean difference BDP/FF/G versus BDP/FF in patient sub-groups. *p < 0.05. *FEV*_*1*_ forced expiratory volume in 1 s, *BDP* beclometasone dipropionate, *FF* formoterol fumarate, *G* glycopyrronium, *BMI* body mass index

**Fig. 2:**
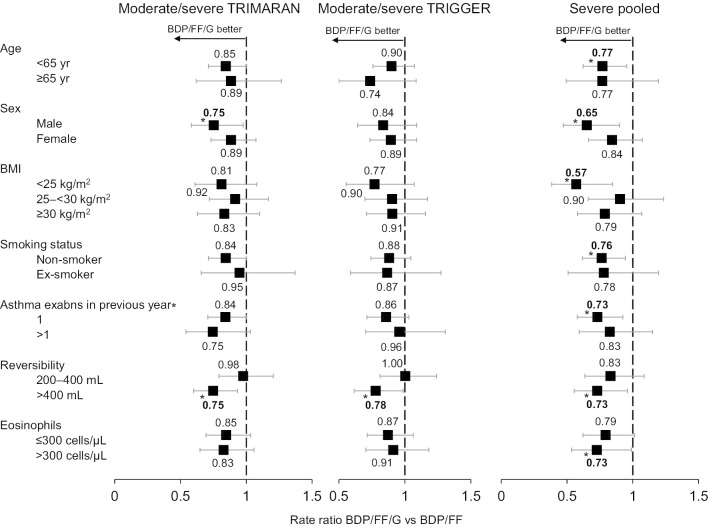
Moderate-to-severe and severe exacerbations, adjusted rate ratio BDP/FF/G versus BDP/FF in patient sub-groups. During the studies, moderate-to-severe exacerbations were experienced by 58.6% and 66.0% patients in the BDP/FF/G and BDP/FF groups, respectively, in TRIMARAN, and by 56.6% and 63.7%, respectively, in TRIGGER; severe exacerbations were experienced by 18.2% and 22.4% patients, respectively, in the pooled population. *p < 0.05. *BDP* beclometasone dipropionate, *FF* formoterol fumarate, *G* glycopyrronium, *BMI* body mass index

**Fig. 3:**
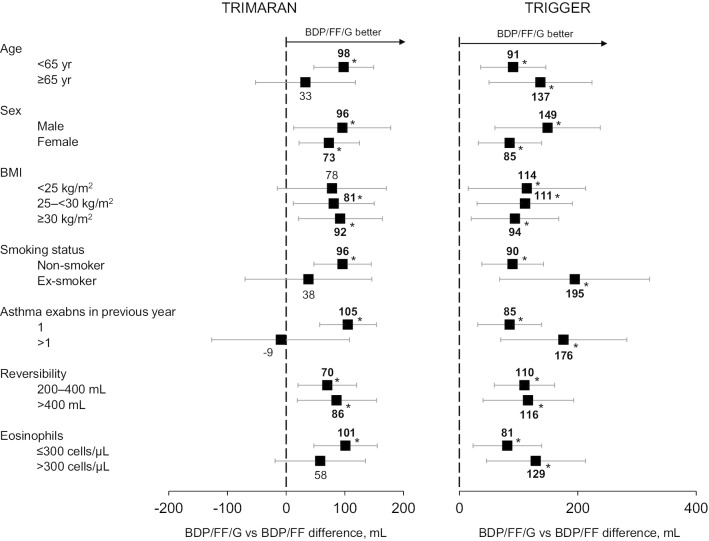
Change from baseline in peak FEV_1_ at Week 26, adjusted least squares mean difference BDP/FF/G versus BDP/FF in patient sub-groups. *p < 0.05. *FEV*_*1*_ forced expiratory volume in 1 s, *BDP* beclometasone dipropionate, *FF* formoterol fumarate, *G* glycopyrronium, *BMI* body mass index

**Fig. 4:**
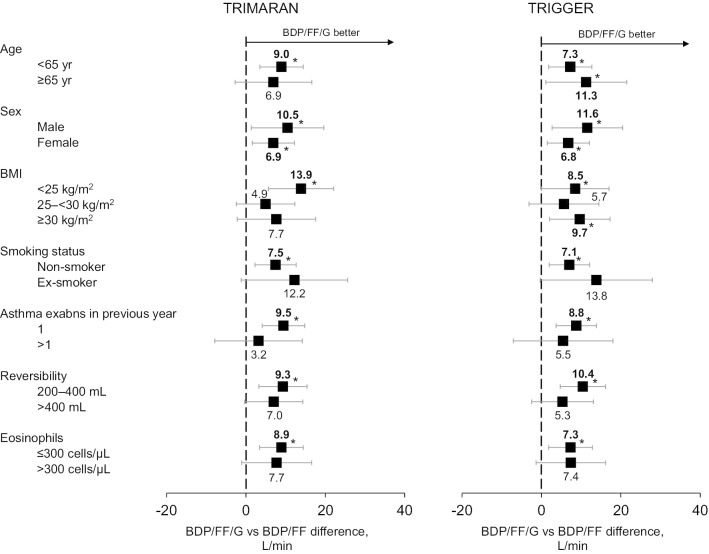
Change from baseline in average morning PEF over the first 26 weeks, adjusted least squares mean difference BDP/FF/G versus BDP/FF in patient sub-groups. *p < 0.05. *PEF* peak expiratory flowm *BDP* beclometasone dipropionate, *FF* formoterol fumarate, *G* glycopyrronium, *BMI* body mass index

The relative effects of BDP/FF/G versus BDP/FF on pre-dose FEV_1_ at Week 26 are shown in Fig. 1, with moderate-to-severe and severe exacerbations in Fig. 2, peak FEV_1_ at Week 26 in Fig. 3, and average morning PEF over the first 26 weeks in Fig. 4. Age category and sex had no consistent marked impact on the relative effect of BDP/FF/G versus BDP/FF for any of the endpoints, although in TRIMARAN lung function efficacy was slightly lower in the ≥ 65 year age group. Smoking status had limited impact, with the lower number of ex-smokers than non-smokers reflected in wider confidence intervals (marked differences between subgroups in patient numbers, as was the case here, are a particular challenge when performing subgroup analyses such as these). Furthermore, BMI categories had no effect on the endpoints apart from severe exacerbations, where the relative effect of BDP/FF/G versus BDP/FF was greatest in patients with normal body weight.

The relative efficacy of BDP/FF/G versus BDP/FF on the lung function endpoints was lower in patients with a history of > 1 exacerbation in TRIMARAN, although not in TRIGGER. Asthma exacerbation history had no consistent effect on the moderate-to-severe exacerbations endpoint; the effect on severe exacerbations in the pooled analysis was lower in the > 1 exacerbations sub-group (17% rate reduction; p = NS) than the 1 exacerbation sub-group (27% rate reduction; p < 0.05). However, more than 80% of patients had a history of just 1 exacerbation in the previous 12 months (Table 1). In addition, the rates of severe exacerbations during the studies were lower in the 1 exacerbation sub-group (0.19 [95% CI 0.16, 0.23] and 0.26 [0.22, 0.31] in the BDP/FF/G and BDP/FF groups, respectively) than in the > 1 exacerbation sub-group (0.50 [0.39, 0.63] and 0.60 [0.48, 0.76]).

Finally, reversibility category had no consistent effect on the lung function endpoints, but did impact the exacerbations endpoints: the sub-group with higher reversibility gained greatest relative benefit from BDP/FF/G compared to BDP/FF, with significant 25% and 22% rate reductions for moderate-to-severe exacerbations in TRIMARAN and TRIGGER, respectively, and a significant 27% rate reduction for severe exacerbations in the pooled analysis.

### Post-hoc analyses by eosinophil levels

At the screening evaluation, approximately 60% of patients in both studies had blood eosinophil levels ≤ 300 cells/µL (Table 1). In TRIMARAN, the greatest relative effect of BDP/FF/G versus BDP/FF on the three lung function endpoints was in the ≤ 300 cells/µL sub-group (Figs. 1, 3, 4). However, in TRIGGER, eosinophil levels did not markedly influence the relative efficacy of BDP/FF/G versus BDP/FF on any of these endpoints.

**Fig. 5:**
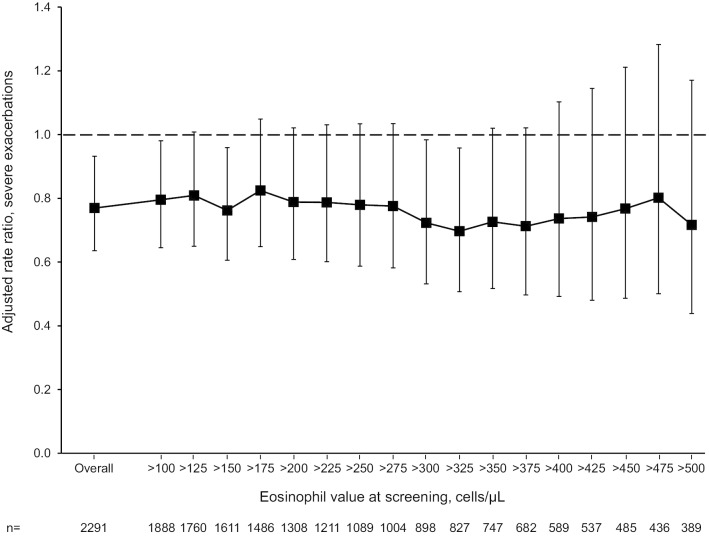
Severe exacerbations, adjusted rate ratio BDP/FF/G versus BDP/FF (pooled analysis) across eosinophil values. Analysed using a negative binomial model including treatment, country, number of exacerbations in the previous year (1 or > 1) and study as fixed effects, and log-time on study as offset. *BDP* beclometasone dipropionate, *FF* formoterol fumarate, *G* glycopyrronium

In the exacerbations analyses, eosinophil levels categorised using the 300 cells/µL cut-off did not influence relative efficacy, either for moderate-to-severe or severe exacerbations (Fig. 2). Similarly, when pooled severe exacerbations were analysed across the range of values, eosinophil levels did not markedly influence the relative efficacy of BDP/FF/G versus BDP/FF, with no clear trend in rate ratios across eosinophil values compared to the overall result, either overall (Fig. 5, with the individual treatment groups in Additional File 1: Fig. S1) or in the sub-group of patients with > 1 exacerbation in the previous year (Additional File 1: Figs. S2 and S3).

## Discussion

In these analyses, most of which were pre-specified, the relative efficacy of extrafine BDP/FF/G versus BDP/FF was consistent and independent of the majority of baseline characteristics. However, in all three exacerbations analyses the relative efficacy of BDP/FF/G versus BDP/FF was consistently greater in patients with higher reversibility compared to those with less reversibility. The post-hoc eosinophil analyses showed no influence on treatment differences for exacerbations, supportive of the broad utility of triple therapy over ICS/LABA.

The moderate-to-severe exacerbations analyses in both TRIGGER and TRIMARAN, and the pooled severe exacerbations analysis all showed that the relative efficacy of BDP/FF/G versus BDP/FF was greater in patients with a higher degree of reversibility. We previously reported post-hoc analyses of TRIMARAN and TRIGGER in the sub-group of patients with persistent airflow limitation, defined as post-bronchodilator FEV_1_ ≤ 80% of the predicted normal value, and a post-bronchodilator ratio of FEV_1_ to forced vital capacity ≤ 0.7 [19]. The results suggested a greater effect for BDP/FF/G in the persistent airflow limitation group, including effects on exacerbations. Overall, this previous analysis of the persistent airflow limitation group coupled with the current reversibility analysis suggests that baseline lung function measurements provide a potential guide to which patients may experience a greater benefit from BDP/FF/G compared to BDP/FF treatment. The finding that the degree of reversibility influences the relative efficacy of BDP/FF/G vs BDP/FF on exacerbations differs from a previous analysis of the effect of the addition of tiotropium to ICS/LABA, in which tiotropium was equally effective regardless of reversibility [20]. However, in contrast to TRIMARAN and TRIGGER, the studies used as the source for these tiotropium data recruited only patients with persistent airflow limitation, and had no baseline reversibility requirements (except to verify the diagnosis of asthma) [4]. Furthermore, the tiotropium reversibility categories were defined on the basis of a 12% and 200 mL threshold, and the only exacerbations endpoint analysed was time to first severe exacerbation. These differences make comparing the results very difficult.

While the degree of reversibility influenced relative efficacy of BDP/FF/G versus BDP/FF on exacerbations, there was no consistent influence on the FEV_1_ and PEF treatment differences. The mechanisms by which the addition of LAMA may cause similar changes in lung function but a different effect on exacerbations according to reversibility status remains unclear. Perhaps more reversible patients have greater day-to-day variability of lung function, and additional bronchodilator treatment in these patients helps stabilise airway smooth muscle tone. Indeed, greater variability in PEF indicates poor asthma control [1], and predicts increased risk of exacerbations [21], with the addition of a LABA to ICS therapy reducing variability in PEF to a greater extent than doubling ICS dose [22]. That said, overall in the current analyses there was a high response on peak FEV_1_ (with BDP/FF/G versus BDP/FF differences of approximately 100 mL in most sub-groups), and a relatively small response on PEF (differences typically < 10 L/min), and as a result neither endpoint helped to clearly identify differential response.

Although some of the baseline clinical characteristics influenced relative efficacy on the lung function endpoints in either TRIMARAN or TRIGGER, there was often a lack of consistency between the two studies. A contributor to this inconsistency is the reduced sample sizes in sub-group analyses. In this context, the consistent relationship between reversibility status and treatment difference on exacerbations in three analyses indicates that this particular relationship is not a chance finding. In contrast, many other differences between sub-groups were inconsistent and small, and therefore unlikely to be clinically relevant. For example, the relative efficacy of BDP/FF/G versus BDP/FF on the lung function endpoints was lower in patients with a history of > 1 exacerbation in TRIMARAN but not in TRIGGER. There are no clear explanations for this. These findings of the lack of effect of the clinical characteristics on lung function endpoints are consistent with previous analyses evaluating the relative effect of inhaled triple therapy versus ICS/LABA alone [20, 23, 24].

It appeared that the benefit of BDP/FF/G was most marked for severe exacerbations in patients with normal body weight. A treatment benefit stratified by weight was not consistently seen for lung function endpoints or moderate to severe exacerbations. The reasons underlying a potential association between weight and treatment differences for severe exacerbations only are unclear.

Eosinophil levels are known to predict the response to corticosteroids in patients with asthma [25], and eosinophil levels can be used to guide therapy, especially in patients who were previously steroid naïve [26–28]. Furthermore, increased eosinophil levels in patients with asthma (especially severe asthma) correlate with increased exacerbation risk [29]. A number of previous analyses have demonstrated that eosinophil levels are not a predictor of response to tiotropium in patients with asthma, in terms of lung function, asthma control, or time to first exacerbation [30–33], although these analyses have not evaluated the impact of eosinophil levels on the efficacy of a LAMA in terms of exacerbation rates. In our analyses, screening blood eosinophil levels did not markedly influence the relative efficacy of BDP/FF/G versus BDP/FF on the rate of exacerbations, either when analysed using a single cut-off or across a continuum, so confirming and extending these previous findings.

There are some limitations to these analyses. They are unpowered, sub-group comparisons, although most were pre-specified. In addition, as indicated by the low rates, few patients experienced severe exacerbations during each study. Furthermore, no inferences can be drawn over cause and effect. As with all such sub-group analyses, the results therefore need to be confirmed in suitably designed prospective clinical trials.

In conclusion, in the patients recruited into TRIMARAN and TRIGGER, all of whom had asthma that was uncontrolled with an ICS/LABA combination, and had a history of at least one exacerbation in the previous year, some selected patient sub-groups gained additional benefit from BDP/FF/G for certain endpoints, supporting the potential for personalization of care. In particular, the relative efficacy of BDP/FF/G versus BDP/FF was consistently greater in more reversible patients than in those with less reversibility in all three exacerbations analyses. However, the relative efficacy of extrafine BDP/FF/G versus BDP/FF was not influenced by a range of other clinical characteristics, thus supporting the broad utility of triple therapy over ICS/LABA in this patient population.

## Supplementary information

**Additional file 1:** **Figure S1**. Severe exacerbations adjusted rates for BDP/FF/G and BDP/FF (pooled analysis) across eosinophil values. **Figure S2.** Severe exacerbations adjusted rate ratio BDP/FF/G versus BDP/FF (pooled analysis) across eosinophil values in the sub-group of patients with > 1 exacerbation in the previous year. **Figure S3.** Severe exacerbations adjusted rates for BDP/FF/G and BDP/FF (pooled analysis) across eosinophil values in the sub-group of patients with > 1 exacerbation in the previous year

## Data Availability

Chiesi commits to sharing with qualified scientific and medical Researchers, conducting legitimate research, patient-level data**,** study-level data**,** the clinical protocol and the full clinical study report of Chiesi Farmaceutici S.p.A.-sponsored interventional clinical trials in patients for medicines and indications approved by the European Medicines Agency and/or the US Food and Drug Administration after *1st January 2015*, following the approval of any received research proposal and the signature of a Data Sharing Agreement. Chiesi provides access to clinical trial information consistently with the principle of safeguarding commercially confidential information and patient privacy. Other information on Chiesi’s data sharing commitment, access and research request’s approval process are available in the Clinical Trial Transparency section of https://www.chiesi.com/en/research-and-development/.

## Abbreviations

BDP: Beclometasone dipropionate
BID: Twice daily
BMI: Body mass index
CI: Confidence interval
FEV_1_: Forced expiratory volume in 1 s
FF: Formoterol fumarate
G: Glycopyrronium
ICS: Inhaled corticosteroid
ITT: Intention-to-treat
LABA: Long-acting β_2_-agonist
LAMA: Long-acting muscarinic antagonist
PEF: Peak expiratory flow
pMDI: Pressurised metered-dose inhaler
