# Supplementary material for: Determinants of response to inhaled extrafine triple therapy in asthma: analyses of TRIMARAN and TRIGGER
Source: Respir Res. 2020 Oct 29;21:285. doi: 10.1186/s12931-020-01558-y (PMC7597025; doi:10.1186/s12931-020-01558-y)
Supplement: Supplementary file 1 — Additional file 1: Figure S1. Severe exacerbations adjusted rates for BDP/FF/G and BDP/FF (pooled analysis) across eosinophil values. Figure S2. Severe exacerbations adjusted rate ratio BDP/FF/G versus BDP/FF (pooled analysis) across eosinophil values in the sub-group of patients with > 1 exacerbation in the previous year. Figure S3. Severe exacerbations adjusted rates for BDP/FF/G and BDP/FF (pooled analysis) across eosinophil values in the sub-group of patients with > 1 exacerbation in the previous year [file 12931_2020_1558_MOESM1_ESM.docx]

# Determinants of response to inhaled extrafine triple therapy in asthma: analyses of TRIMARAN and TRIGGER

Dave Singh, Johann Christian Virchow, Giorgio Walter Canonica, Andrea Vele, Maxim Kots, George Georges, Alberto Papi

# Additional file 1

Figure S1. Severe exacerbations adjusted rates for BDP/FF/G and BDP/FF (pooled analysis) across eosinophil values.


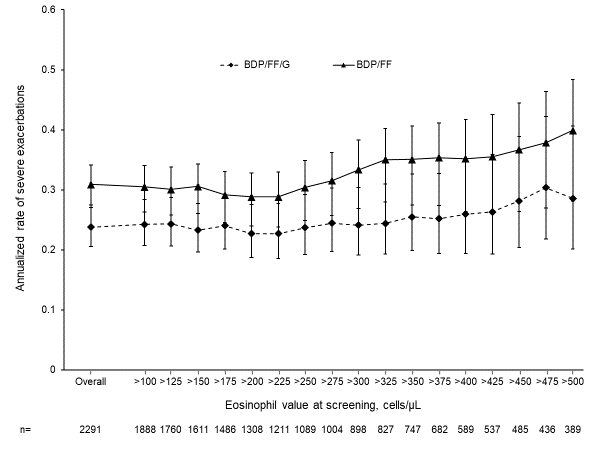


Analysed using a negative binomial model including treatment, country, number of exacerbations in the previous year (1 or >1) and study as fixed effects, and log-time on study as offset. BDP: beclometasone dipropionate; FF: formoterol fumarate; G: glycopyrronium.

Figure S2. Severe exacerbations adjusted rate ratio BDP/FF/G versus BDP/FF (pooled analysis) across eosinophil values in the sub-group of patients with >1 exacerbation in the previous year.


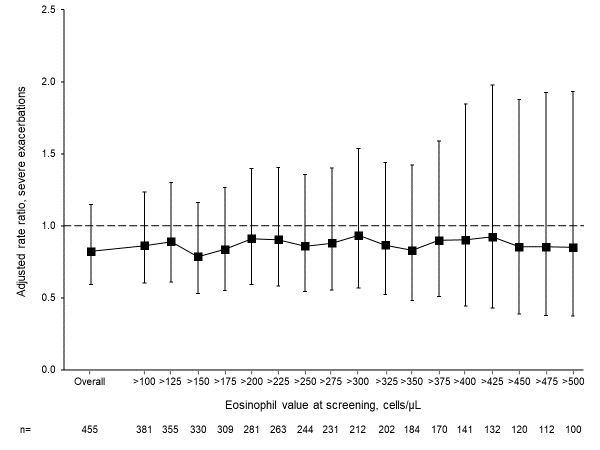


Analysed using a negative binomial model including treatment, country and study as fixed effects, and log-time on study as offset. BDP: beclometasone dipropionate; FF: formoterol fumarate; G: glycopyrronium.

Figure S3. Severe exacerbations adjusted rates for BDP/FF/G and BDP/FF (pooled analysis) across eosinophil values in the sub-group of patients with >1 exacerbation in the previous year.


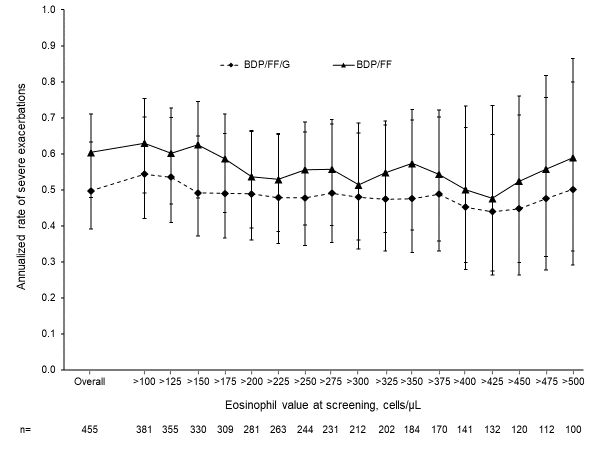


Analysed using a negative binomial model including treatment, country and study as fixed effects, and log-time on study as offset. BDP: beclometasone dipropionate; FF: formoterol fumarate; G: glycopyrronium.
